# Single nucleotide polymorphisms of nucleotide excision repair and homologous recombination repair pathways and their role in the risk of osteosarcoma

**DOI:** 10.12669/pjms.312.6569

**Published:** 2015

**Authors:** Guojun Jin, Min Wang, Weida Chen, Wei Shi, Jiapeng Yin, Wang Gang

**Affiliations:** 1 Guojun Jin, Department of Hand and foot Surgery, Taizhou First People’s Hospital, Taizhou, China; 2 Min Wang, Department of Orthopedics, Taizhou First People’s Hospital, Taizhou, China; 3 Weida Chen, Department of Hand and foot Surgery, Taizhou First People’s Hospital, Taizhou, China; 4 Wei Shi, Department of Hand and foot Surgery, Taizhou First People’s Hospital, Taizhou, China; 5 Jiapeng Yin, Department of Burns and Plastic Surgery, First Affiliated Hospital of Xiamen University, Xiamen, China; 6 Wang Gang, Department of Hand and foot Surgery, Taizhou First People’s Hospital, Taizhou, China

**Keywords:** Single Nucleotide Polymorphism, Osteosarcoma, Nucleotide excision repair, Homologous recombination repair

## Abstract

**Objective:**

To evaluate the influence of polymorphisms in nucleotide excision repair (NER) and homologous recombination repair (HRR) pathways on the development of osteosarcoma patients.

**Methods:**

Genotypes of ERCC1 rs11615 and rs3212986, ERCC2 rs1799793 and rs13181, NBN rs709816 and rs1805794, RAD51 rs1801320, rs1801321 and rs12593359, and XRCC3 rs861539 were conducted by Polymerase Chain Reaction Restriction Fragment Length Polymorphism (PCR-RFLP) assay.

**Results:**

Total 148 osteosarcoma patients and 296 control subjects were collected from Taizhou First People’s Hospital. Conditional logistic regression analyses found that individuals carrying with GA+AA genotype of ERCC2 rs1799793 and GC+CC genotype of NBN rs1805794 were significantly associated with increased risk of osteosarcoma, and the ORs(95%CI) were 1.58(1.03-2.41) and 2.66(1.73-4.08), respectively. We found that GA+AA genotype of ERCC2 rs1799793 or GC+CC genotype of NBN rs1805794 were associated with an increased risk of osteosarcoma in females, with ORs(95%CI) of 2.42(1.20-4.87) and 2.01(1.07-4.23), respectively.

**Conclusion:**

Our results suggest that ERCC2 rs1799793 and NBN rs1805794 polymorphisms were associated with an increased risk for osteosarcoma, which suggests that NER and HRR pathways modulate the risk of developing osteosarcoma.

## INTRODUCTION

Osteosarcoma is a rare bone cancer that has a first peak of incidence in adolescents around 16 years, and a second peak in patients older than 60 years.^1^ Osteosarcoma is a kind of disease which caused by complex, multistep, and multifactorial process.^2^ Previous studies have investigated the cancer stem cells and their potential to cause tumors, and genetic factors play an important role in the development of osteosarcoma, and several studies showed that DNA repaired genes are involved in the pathogenesis of this cancer.^3^,^4^

Genetic polymorphisms in DNA repair genes may lead to amino acid substitution and cause differential capacity to repair DNA damage. The genetic polymorphisms in DNA repair genes have been found to be associated with increased genetic instability and carcinogenesis.^5^ In mammalian cells, there were four different DNA repair mechanisms, including nucleotide excision repair (NER), homologous recombination repair (HRR), double-strand break repair (DSBR) and mismatch repair.^6^,^7^ All these DNA repair pathways are finely regulated for the maintenance of genomic integrity and modulation of repair capacity in response to DNA damage and thus susceptibility to cancer risk.

Excision repair cross-complementation group 2 (ERCC2) gene and excision repair cross complementation group 1 (ERCC1) are parts of an endonuclease complex. More complex DNA damage requires different mechanisms such as HRR for successful repair. In HRR, nibrin (NBN) is part of the complex involved in recognition of DNA damage, while RAD51 recombinase (RAD51) catalyses homologous search and strand invasion with the help of other proteins, including X-ray complementing defective repair in Chinese hamster cells 3 (XRCC3).^8^,^9^

Single nucleotide polymorphisms (SNPs) of NER genes have already been associated with development of different cancer types,^3^,^4^,^10^-^15^ including osteosarcoma,^14^,^15^ but all results are not concordant. On the other hand, only a few studies have investigated the influence of HRR SNPs on the development of osteosarcoma.^3^,^4^ The aim of the present study was to evaluate the influence of polymorphisms in NER and HRR pathways on the development of osteosarcoma patients.

## METHODS

### Patients

Between January 2012 and January 2014, 148 osteosarcoma patients were diagnosed from Taizhou First People’s Hospital. Osteosarcoma patients were newly diagnosed and histopathologically confirmed independently by two gynecologic pathologists. 296 control subjects were collected from health check center of Taizhou First People’s Hospital, who came to our hospital for medical check up. All the control subjects were confirmed not suffering from osteosarcoma, and had no medical history of any tumor or cancer and no family history of osteosarcoma or other cancers in first-degree relatives. The control subjects were matched to cases by sex and age, and two cases were matched to one case. The study was approved by the Taizhou First People’s Hospital.

Controls were investigated to obtain demographic parameters. For the cases, clinical and pathological information were extracted from the medical records, including tumor stage and location, histological type, and family history of cancer.

### Blood samples and genotyping

5ml peripheral blood was collected from each patient and was kept in -70ºC until use. Genomic DNA was isolated from peripheral blood using the TIANamp Blood DNA Kit (Tiangen, Beijing, China) according to the manufacturer’s instructions. Genotypes of ERCC1 rs11615 and rs3212986, ERCC2 rs1799793 and rs13181, NBN rs709816 and rs1805794, RAD51 rs1801320, rs1801321 and rs12593359, and XRCC3 rs861539 were conducted by Polymerase Chain Reaction Restriction Fragment Length Polymorphism (PCR-RFLP) assay. Probes and primers for ERCC1 rs11615 and rs3212986, ERCC2 rs1799793 and rs13181, NBN rs709816 and rs1063054, RAD51 rs1801320, rs1801321 and rs12593359, and XRCC3 rs861539 were designed using Primer 5.0 software (PREMIER Biosoft, Palo Alto, CA). The cycling programme of PCR reaction condition was performed as follows: one initial denaturation step at 5 minutes at 94ºC, followed by 35 denaturation cycles of 45s at 94ºC, 60s of annealing at 62ºC, and 60s of extension at 72ºC, and followed by a final extension at 72ºC for 10 minutes. In order to perform quality control, genotyping was repeated in 20% samples to check for genotyping accuracy.

### Statistical analysis

Continuous variables were expressed as the mean ± SD and analyzed by student t test. Categorical variables were shown as N(%) and analyzed by χ^2^-test. The χ^2^-test was performed to verify whether the genotype distributions were in Hardy–Weinberg equilibriums of ERCC1 rs11615 and rs3212986, ERCC2 rs1799793 and rs13181, NBN rs709816 and rs1805794, RAD51 rs1801320, rs1801321 and rs12593359, and XRCC3 rs861539. The association between osteosarcoma group and control group were assessed using unconditional logistic regression, and the results were expressed using odds ratio (OR) and their confidence intervals (CI). All *P*-values were two sided, and a *P*-value was regarded as statistically significant when it less than 0.05.

## RESULTS

The characteristics of patients with osteosarcoma and controls are shown in Table-I. We did not find significant difference between cases and controls for the frequency-matched variables gender and age at interview (P>0.05). The mean ages of patients with osteosarcoma and controls were 17.7±8.2 and 19.2±5.3 years, respectively. There was no significant difference between cases and controls in terms of family history of cancer (P>0.05). For the stage of osteosarcoma, 106 (71.62%) were at stage of I-II, and 42(28.38%) were at stage of III. For the tumor location of these cases, 107(72.30%) were extremities, and 41(27.70%) located at other places.

**Table-I:** Characteristics of patients with osteosarcoma and controls.

Characteristics	Cases	%	Controls	%	OR(95%CI)	P value
*Gender*
Male	91	61.49	181	61.15	1.0(Ref.)	
Female	57	38.51	115	38.85	0.98(0.64-1.51)	0.95
Mean age, years	17.7±8.2		19.2±5.3			
<20	87	58.78	164	55.41	1.0(Ref.)	
≥20	61	41.22	132	44.59	0.87(0.57-1.32)	0.5
*Family history of cancer*
Positive	35	23.65	63	21.28	1.0(Ref.)	
Negative	113	76.35	233	78.72	0.87(0.53-1.44)	0.57
*Stage*
I-II	106	71.62				
III	42	28.38				
*Histological type*
Osteoblastic	48	32.43				
Chondroblastic	70	47.3				
Other	30	20.27				
Tumor location
Extremities	107	72.3				
Other	41	27.7				

By χ^2^-test, genotype distributions of ERCC1 rs11615 and rs3212986, ERCC2 rs1799793 and rs13181, NBN rs709816 and rs1805794, RAD51 rs1801320, rs1801321 and rs12593359, and XRCC3 rs861539 in the control subjects were found to be in line with Hardy–Weinberg equilibrium. Conditional logistic regression analyses found that individuals carrying with GA+AA genotype of ERCC2 rs1799793 were significantly associated with increased risk of osteosarcoma when compared with GG genotype (OR=1.58, 95%CI=1.03-2.41). Moreover, individuals carrying GC+CC genotype of NBN rs1805794 were correlated with a heavy enhanced risk of osteosarcoma when compared with GG genotype, and the OR(95%CI) was 2.66(1.73-4.08). However, we did not find significant association between ERCC1 rs11615 and rs3212986, ERCC2 rs13181, NBN rs709816, RAD51 rs1801320, rs1801321 and rs12593359, and XRCC3 rs861539 and risk of osteosarcoma.

**Table-II:** Genotype distribution of ten SNPs in NER and HRR pathways between cases and controls.

Gene	Genotype	Cases	%	Controls	%	OR(95%CI)^1^	P value
ERCC1 rs11615	TT	63	42.57	135	45.3	1.0(Ref.)	-
	TC+CC	85	57.43	163	54.7	1.12(0.74-1.70)	0.58
ERCC1 rs3212986	GG	79	53.38	169	56.71	1.0(Ref.)	-
	GA+AA	69	46.62	129	43.29	1.14(0.75-1.73)	0.5
ERCC2 rs1799793	GG	84	56.76	201	67.45	1.0(Ref.)	-
	GA+AA	64	43.24	97	32.55	1.58(1.03-2.41)	0.03
ERCC2 rs13181	AA	86	58.11	181	60.74	1.0(Ref.)	-
	AC+CC	62	41.89	117	39.26	1.12(0.73-1.70)	0.59
NBN rs709816	GG	55	37.16	115	38.59	1.0(Ref.)	-
	GC+CC	93	62.84	183	61.41	1.06(0.69-1.63)	0.78
NBN rs1805794	GG	52	35.14	127	42.62	1.0(Ref.)	-
	GC+CC	96	64.86	171	57.38	2.66(1.73-4.08)	<0.001
RAD51 rs1801320	GG	101	68.24	213	71.48	1.0(Ref.)	-
	GC+CC	47	31.76	85	28.52	1.17(0.74-1.82)	0.48
RAD51 rs1801321	GG	88	59.46	187	62.75	1.0(Ref.)	-
	GT+TT	60	40.54	111	37.25	1.15(0.75-1.75)	0.5
RAD51 rs12593359	TT	49	33.11	105	35.23	1.0(Ref.)	-
	TG+GG	99	66.89	193	64.77	1.10(0.71-1.71)	0.66
XRCC3 rs861539	CC	77	52.03	173	58.05	1.0(Ref.)	-
	CT+TT	71	47.97	125	41.95	1.28(0.84-1.93)	0.23

1 Adjusted for sex, age and family history of cancer.

**Table-III:** Stratification analysis on the association between rs699947 and rs2010963 and demographic characteristics in osteosarcoma risk.

Variables	ERCC2 rs1799793		OR(95%CI)	P	NBN rs1805794		OR(95%CI)	P
	Cases/Controls				Cases/Controls	
	GG	GA+AA	GA+AA vs GG		GG	GC+CC	GC+CC vs GG	
Gender								
Male	58/124	33/57	1.23(0.71-2.17)	0.43	35/74	56/107	1.11(0.64-1.92)	0.7
Female	26/77	31/38	2.42(1.20-4.87)	0.007	17/53	40/62	2.01(1.07-4.23)	0.04
Age								
<20	53/34	119/45	1.70(0.94-3.05)	0.06	31/69	56/95	1.31(0.74-2.34)	0.32
≥20	31/82	30/50	1.59(0.82-3.06)	0.14	21/58	40/74	1.49(0.76-2.97)	0.21
Family history of cancer								
Positive	11/28	24/35	1.75(0.68-4.64)	0.21	9/15	26/48	0.90(0.32-2.68)	0.83
Negative	73/173	40/60	1.58(0.94-2.63)	0.06	43/112	70/121	1.51(0.93-2.45)	0.08

Moreover, we conducted stratification analysis on the association between ERCC2 rs1799793 and NBN rs1805794 and demographic characteristics in osteosarcoma patients. We found that GA+AA genotype of ERCC2 rs1799793 or GC+CC genotype of NBN rs1805794 were associated with an increased risk of osteosarcoma in females, and the ORs(95%CI) were 2.42(1.20-4.87) and 2.01(1.07-4.23), respectively.

## DISCUSSION

In this hospital-based case-control study, we investigated the role of ten SNPs in NER and HRR pathways about the risk of osteosarcoma. Since there is increasing evidence that genetic variation leads to different DNA repair capacities in the human population, such common polymorphisms can play a role in an individual’s genetic susceptibility to cancer.^16^ Mutations in DNA repaired gene may lead to decrease or loss of its DNA repair capacity and confer the variation in susceptibility to diverse malignant tumors among individuals. Many studies have indicated that polymorphisms in DNA repair genes may modify the risk for cancer, such as esophageal cancer, breast cancer, glioma and prostate cancer,^17^-^20^ while few studies reported the role of DNA repair genes and development of osteosarcoma.^21^

We also found that ERCC2 rs1799793 and NBN rs1805794 may be associated with carcinogenesis of osteosarcoma. GA+AA genotype of ERCC2 rs1799793 and GC+CC genotype of NBN rs1805794 were associated with increased risk of osteosarcoma, but that gene polymorphisms of ERCC1 rs11615 and rs3212986, ERCC2 rs13181, NBN rs709816, RAD51 rs1801320, rs1801321 and rs12593359, and XRCC3 rs861539 were not correlated with risk of this cancer. Previous studies have showed that the non-synonymous ERCC2 rs1799793 SNP is associated with lower DNA repair capacity.^22^ Our results are in line with the proposed biological effect of ERCC2 rs1799793, as lower repair capacity may cause increased DNA damage and thus increase the risk of osteosarcoma. For NBN rs1805794, previous studies have showed that NBN SNPs can influence the DNA repair capacity, and NBN rs1805794 genotypes confer to differences in levels of DNA damage and osteosarcoma risk.^3^,^4^ Our study is in agreement with previous studies, GC+CC genotype of NBN rs1805794 was associated with increased risk of developing osteosarcoma.

Development of osteosarcoma was caused by different multiple factors, including genetic and environmental factors.^2^,^14^,^15^ Our study showed that GA+AA genotype of ERCC2 rs1799793 or GC+CC genotype of NBN rs1805794 were associated with an increased risk of osteosarcoma in females, which indicated that genetic synergistic effect between ERCC2 rs1799793 and NBN rs1805794 genetic variants and gender increases the risk of cancer. However, no previous studies have investigated the correlation between DNA repair genes and demographic factors on the risk of osteosarcoma, therefore, further studies are greatly needed to confirm our results.

There are some limitations to the present study that should be mentioned. First of all, although this study suggested that ERCC2 rs1799793 and NBN rs1805794 polymorphisms were associated with osteosarcoma risk, more biological background data are needed to explain our results. The current finding might involve gene-to-environment interactions, which were not explored in the present study. Second, the sample size of this study is relatively small, which may not have enough statistical power to explore the real association. Third, this is a hospital based case control study, so the selection bias cannot be ignored and the subjects may not be representative of the general population. Finally, these results should be interpreted with caution because the population was only from China, which reduces the possibility of confounding from ethnicity, so it does not permit extrapolation of the results to other ethnic groups.

In conclusion, our study is, to our knowledge, the first to examine prospectively an increased risk role of ten SNPs in DNA repair pathway in osteosarcoma susceptibility, and we found that ERCC2 rs1799793 and NBN rs1805794 polymorphisms modulate the risk of developing osteosarcoma. Additional studies are greatly needed to confirm this finding.
